# Results from a PI-RADS-based MRI-directed diagnostic pathway for biopsy-naive patients in a non-university hospital

**DOI:** 10.1007/s00261-021-03249-8

**Published:** 2021-08-20

**Authors:** Jeroen S. Reijnen, Jon B. Marthinsen, Alf O. Tysland, Christoph Müller, Irina Schönhardt, Erlend Andersen, Therese Seierstad, Knut H. Hole

**Affiliations:** 1 Department of Radiology, Sørlandet Hospital, Kristiansand, Norway; 2 Institute of Clinical Medicine, University of Oslo, Oslo, Norway; 3 Department of Urology, Sørlandet Hospital, Kristiansand, Norway; 4 Department for Cancer Treatment, Sørlandet Hospital, Kristiansand, Norway; 5 Department of Pathology, Sørlandet Hospital, Kristiansand, Norway; 6 Division of Radiology and Nuclear Medicine, Radiumhospitalet, Oslo University Hospital, P.o.box 4950, Nydalen, Oslo Norway

**Keywords:** Prostate neoplasms, Magnetic resonance imaging, Biopsy, Patient care team, Algorithms

## Abstract

**Purpose:**

To assess the safety and performance of a MRI-directed diagnostic pathway for patients with first-time suspicion of prostate cancer in a non-university hospital.

**Methods:**

Between May 2017 and December 2018 all biopsy-naive patients examined in our hospital followed a MRI-directed diagnostic work-up algorithm based on PI-RADS score. In short, PI-RADS 1–2 was generally not biopsied and PI-RADS 3–5 was reviewed by a multidisciplinary team. Patients with PI-RADS 4-5 were all referred to biopsy, either transrectal ultrasound-guided biopsy or MRI in-bore biopsy for small tumors and for sites difficult to access. PI-RADS scores were compared to the histopathology from biopsies and surgical specimens for patients who had prostatectomy. Non-biopsied patients were referred to a safety net monitoring regimen.

**Results:**

Two hundred and ninety-eight men were enrolled. 97 (33%) had PI-RADS 1–2, 44 (15%) had PI-RADS 3, and 157 (53%) had PI-RADS 4–5. 116 (39%) of the patients avoided biopsy. None of these were diagnosed with significant cancer within 2–3.5 years of safety net monitoring. Almost all high ISUP grade groups (≥ 3) were in the PI-RADS 4–5 category (98%). Prostatectomy specimens and systematic biopsies from MRI-negative areas indicated that very few clinically significant cancers were missed by the MRI-directed diagnostic pathway.

**Conclusion:**

Our findings add to evidence that a MRI-directed diagnostic pathway can be safely established in a non-university hospital. The pathway reduced the number of biopsies and reliably detected the site of the most aggressive cancers.

**Graphic abstract:**

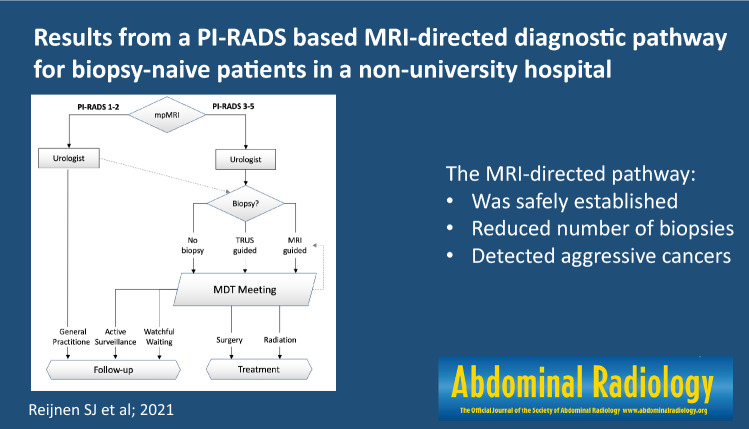

**Supplementary Information:**

The online version contains supplementary material available at 10.1007/s00261-021-03249-8.

## Introduction

Magnetic resonance imaging (MRI) is increasingly used in the diagnostic work-up of biopsy-naive patients with suspected prostate cancer. The ability of MRI to detect and localize clinically significant prostate cancer has been established [1–6] and MRI-directed biopsy strategies have been shown to benefit biopsy-naive patients [7–10]. A recent Cochrane review concluded that MRI-directed diagnostic work-up increased the detection of clinically significant prostate cancer and reduced the detection of insignificant prostate cancer compared to systematic biopsy [11]. On the basis of this body of evidence, the Prostate Imaging-Reporting and Data System (PI-RADS) committee recently proposed the PI-RADS MRI-directed biopsy pathway [12].

PI-RADS is both observer- and experience-dependent [13–16] and the performance of a MRI-directed diagnostic pathway also relies on adequate biopsy targeting [10, 17, 18]. Thus, to achieve widespread adoption of MRI-directed diagnostic pathways without routine systematic biopsies, a large and varied body of knowledge is needed. Most of the data collected so far originate from studies with multicenter design, with the majority of data collected from academic, tertiary referral centers [5, 7, 18]. Data from non-university hospitals are highly warranted [11, 12, 16, 19].

Based on the results of the PROMIS trial [2], we introduced in 2017 a MRI-directed diagnostic pathway for biopsy-naive patients at our non-university hospital. Our pathway is similar to the pathway proposed by the PI-RADS-steering committee in 2019 [12]. The purpose of this study was to report the performance of our MRI-directed diagnostic pathway for patients with first-time suspicion of prostate cancer.

## Materials and methods

### Study cohort and patient workflow

**Fig. 1:**
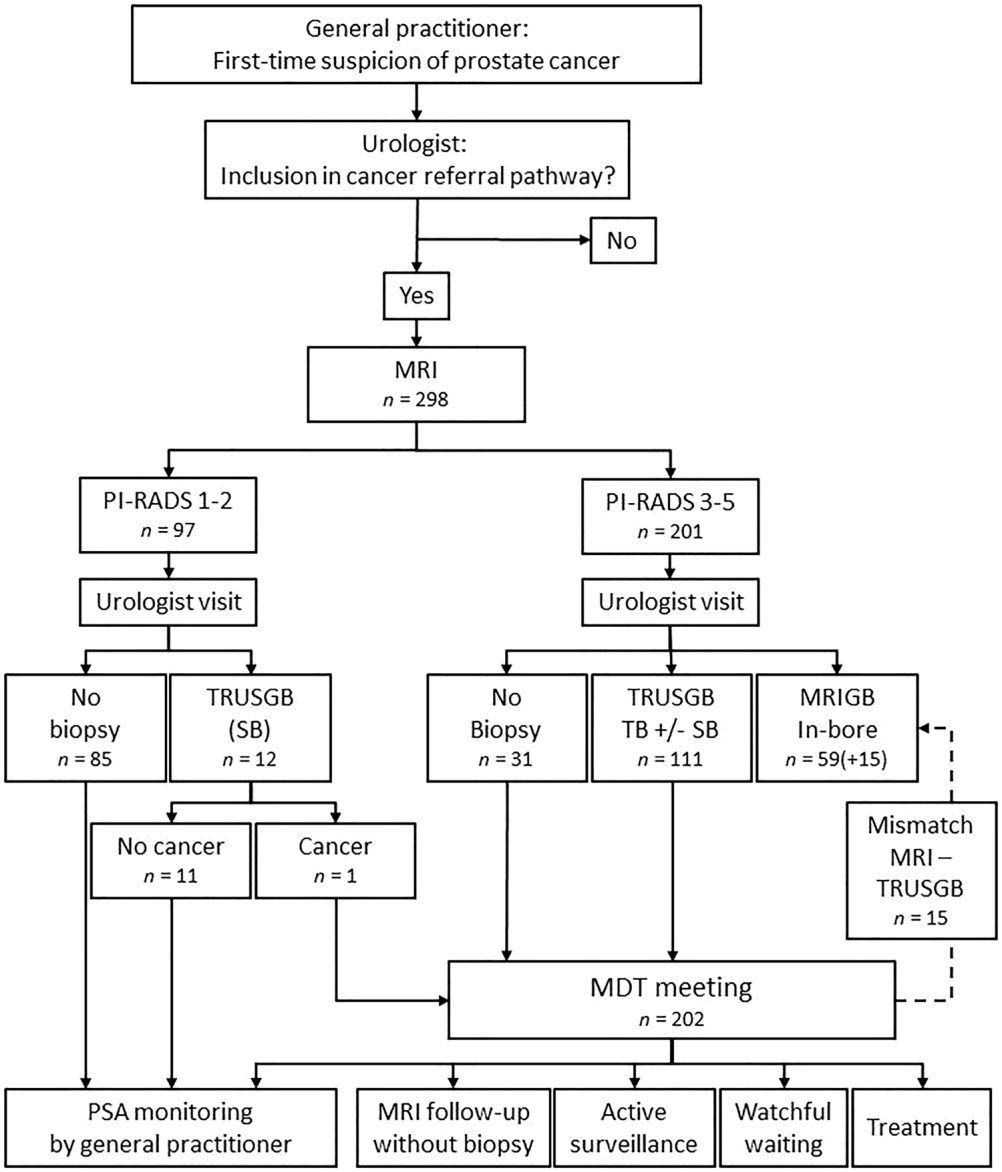
Flow-chart of the MRI-directed pathway for patients with suspicion of prostate cancer referred to our hospital. *TRUSGB* transrectal ultrasound-guided biopsy, *SB* systematic biopsies, *TB* MRI-targeted biopsies with cognitive fusion, *MRIGB* magnetic resonance imaging-guided biopsy, *MDT* multidisciplinary team

Almost all patients with suspected prostate cancer within the local county are referred to the prostate cancer referral pathway at our hospital. The referral population is 187.000. All referrals to our hospital for first-time suspicion of prostate cancer were assessed by an urologist who initiated the pathway. After inclusion into the prostate cancer referral pathway, all patients had MRI prior to biopsy. A flow-chart summarizing the MRI-directed pathway is shown in Fig. 1. The data from these patients were prospectively entered into the Institutional Prostate Cancer Quality Registry. This retrospective single-center study of prospectively collected clinical routine data was approved by the Institutional Review Board that waived the need for informed consent. From May 2017 until December 2018 data from a total of 298 patients were enrolled. The mean age was 61 years (standard deviation 7.6, range 31–75) and the mean prostate-specific antigen (PSA) was 5.9 ng/ml (standard deviation 7.2, range 0.7–40).

### Multiparametric MRI

**Fig. 2:**
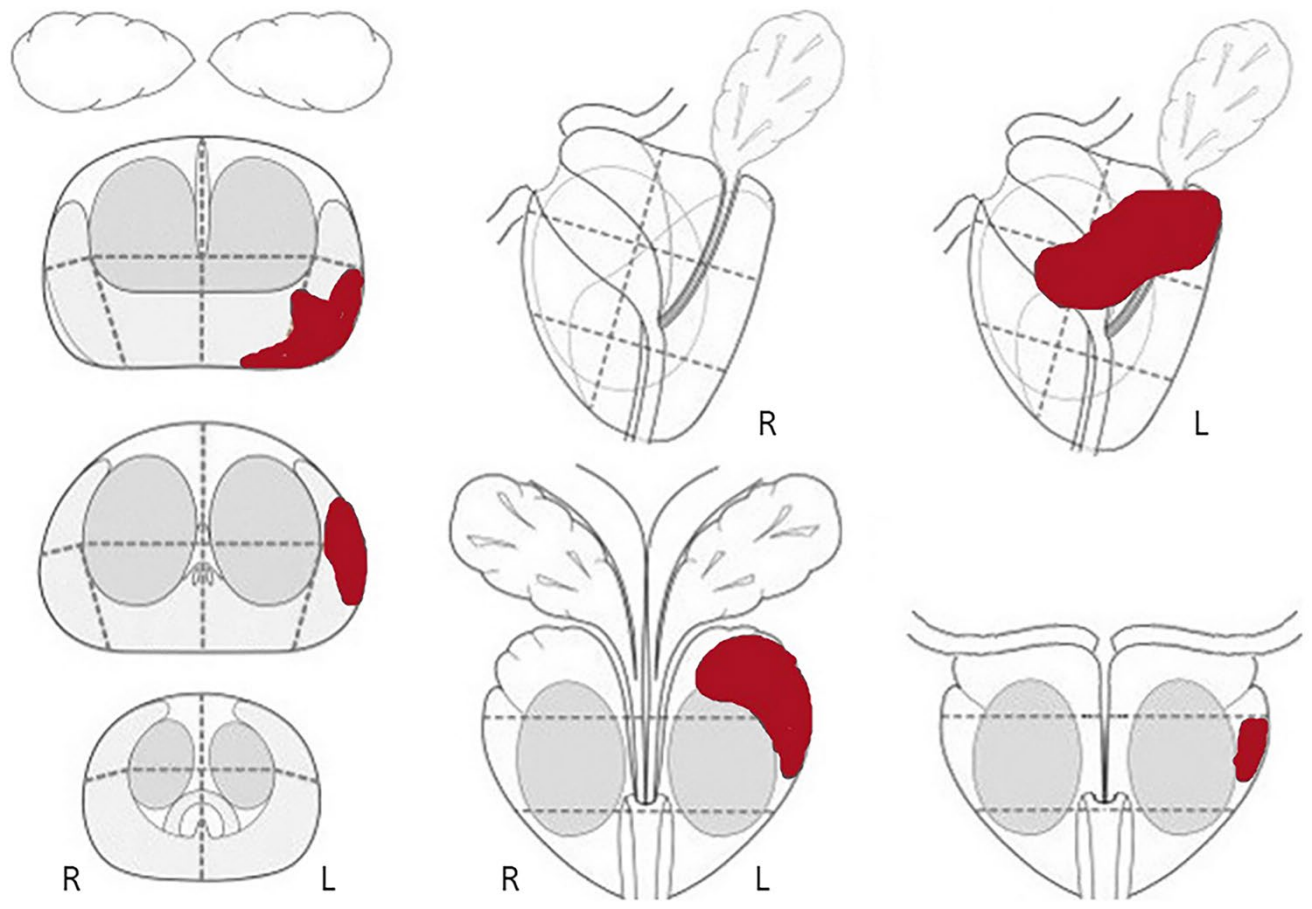
Example of the template drawing of the location and extent of the prostate cancer as depicted by MRI

All patients were examined using a 3 T Siemens MAGNETOM Skyra MRI scanner and phased-array coils. Preparation of the patient included a cleansing enema (toilax 10 mg/5 ml bisakodyl, Orion Corporation) to void the rectum and intravenous administration of 2 ml Buscopan (Boehringer Ingelheim) and intramuscular injection of 1 mg Glucagon (Novo Nordisk A/S) to reduce spasmodic activity. The MRI protocol included morphological T1- and T2-weighted images as well as diffusion-weighted images and dynamic contrast-enhanced (DCE) images. The MRI sequences and acquisition parameters are summarized in Online Resource 1. The image quality complies with the technical requirements from PI-RADS v2 and 2.1 [16, 20, 21]. DCE imaging was performed after *i.v.* injection of gadoterate meglumine (DOTAREM, Guerbet LLC,) at a dose of 0.1 mmol/kg body weight at a rate of 2 ml/s followed by a 20 ml saline flush. All MRI examinations were independently interpreted by one of two experienced radiologists (JSR, JBM) with 10 and 5 years of experience with prostate MRI, respectively. The results from the clinical exam by the general practitioner and serum PSA level were known to the readers when interpreting the images. For each patient, up to three lesions were assigned a score according to the PI-RADS v2 [20]. The location and extent of the lesions were drawn in the PI-RADS report template (Fig. 2).

### Prostate biopsy

Based on clinical information, PI-RADS score, and a template drawing of tumor localization and extent, the urologist decided the next step and the need for biopsy. For patients with PI-RADS 1–2 no biopsy was performed unless risk factors were present (PSA metrics, digital rectal examination findings, family history, comorbidity, and life expectancy). Systematic transrectal ultrasound (TRUS) biopsies were obtained for patients with risk factors. For patients with PI-RADS 3 the decision on whether to biopsy was made by the multidisciplinary team (MDT) based on MRI and risk factors. Patients with PI-RADS 4–5 were all referred to biopsy, either TRUS biopsies or MRI in-bore biopsy for small tumors and for sites difficult to access. Patients with TRUS-guided biopsies had targeted biopsies and, in most cases, also 12-core systematic biopsies. In some patients with large tumors at MRI, systematic biopsies were omitted. The systematic biopsies were obtained according to a standardized template that allowed correlation to MRI findings. Targeting was performed using cognitive fusion based on the template drawing (Fig. 2).

Transrectal in-bore MRI-guided biopsies were performed by one of the two radiologists or in collaboration, using the same scanner as for diagnostic MRI. Robotic assistance (Remote Controlled Manipulator, Soteria Medical) was used for steering the needle guide (Invivo). Two to four biopsies per target lesion were obtained with an 18G biopsy needle (Tru-Core II, Argon Medical Devices). The biopsy specimens were fixed in 10% buffered formalin, embedded in paraffin, and stained with hematoxylin and eosin (HE), according to our standard hospital protocol. An experienced uropathologist (IS) evaluated the biopsies and assigned a grade group according to the International Society of Urological Pathology (ISUP) criteria [22]. Clinically significant cancer was defined as Gleason score of at least 3 + 4 (ISUP grade group ≥ 2) [12, 22].

### Multidisciplinary team and safety net

The prostate MDT consisted of urologists, radiologists, oncologists, and a pathologist and met weekly. All PI-RADS 3–5 and PI-RADS 1–2 with positive biopsy were discussed (Fig. 1). Further, the urologist had the option to consult the MDT when in doubt on whether to biopsy a patient with PI-RADS 1–2. If MDT suspected that the biopsy findings were not representative, i.e., mismatch between findings at MRI and the results from TRUS biopsies, patients were referred to additional targeted in-bore MRI biopsy. For patients with detected prostate cancer the MDT decided whether the patient should undergo treatment or be subjected to follow-up (active surveillance or watchful waiting).

A safety net was established for non-biopsied patients. Non-biopsied PI-RADS 1–2 patients were referred to follow-up by the GP with instruction to contact the urologist at our hospital if the PSA metrics increased above a threshold value. Non-biopsied patients with PI-RADS 3 were followed up by PSA measurements mainly at six-month intervals at the hospital (12 of 21) or by the GP (9 of 21). Five of the patients followed at the hospital had a new MRI and three were biopsied later in the follow-up period without clinically significant cancer. It is important to point out that the public health care system in our region is organized so that all patients will be referred back to our hospital exclusively. This, we consider an important part of the oncologic safety.

### Data analyses

Descriptive statistics are used to present our experiences with the MRI-directed prostate pathway. PI-RADS scores, per patient and per lesion, were compared to the histopathology from biopsy and surgical specimens for patients who had prostatectomy. PI-RADS scores 1–2 and scores 4–5 were grouped for the data analysis. For the analyses per patient, the highest overall PI-RADS score for each patient was used.

## Results

**Fig. 3:**
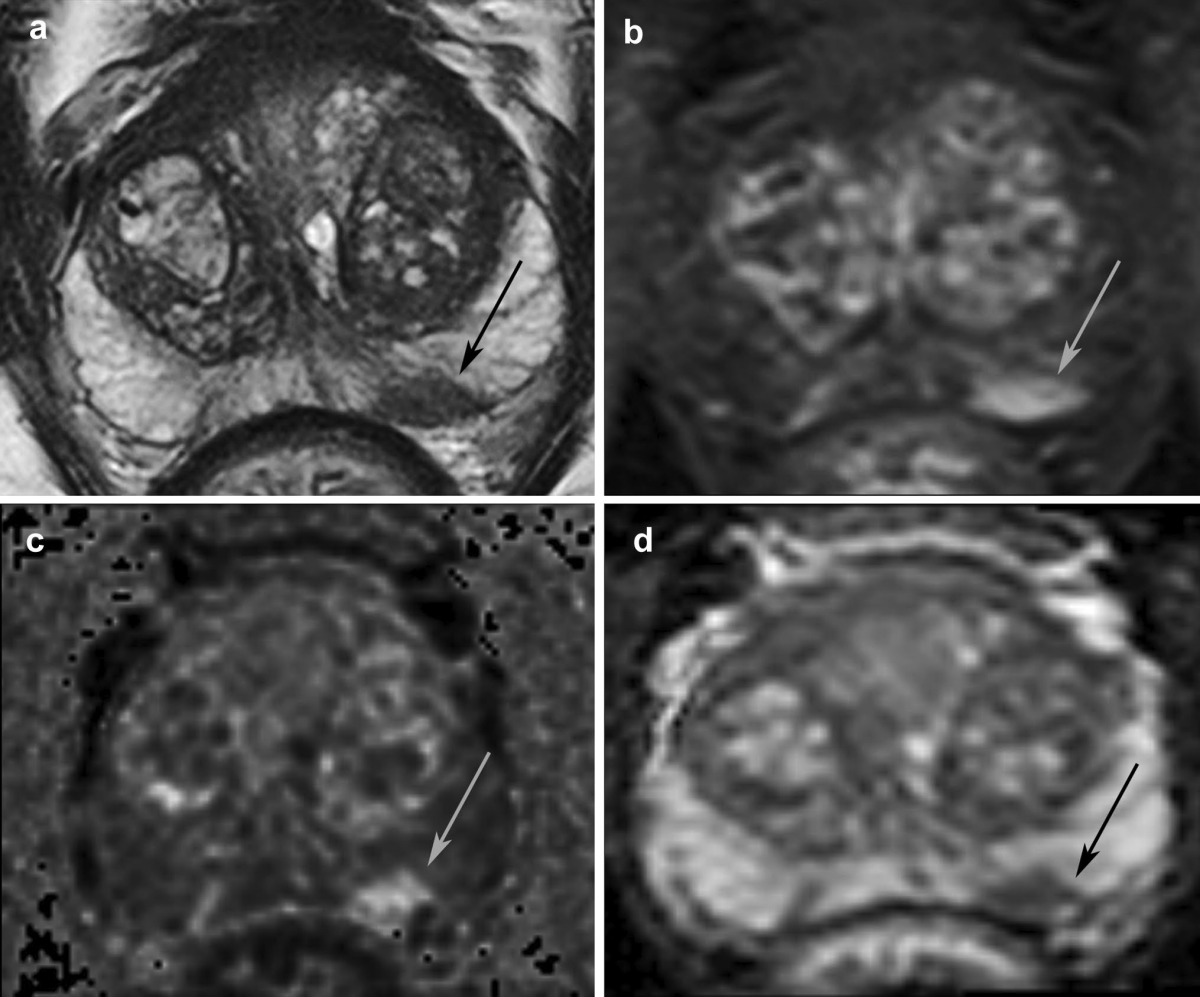
Representative MRI images used in our MRI-directed diagnostic pathway for prostate cancer. 60-year-old man with an ISUP grade group 2 cancer posterolaterally in the peripheral zone left side (arrow). **a** Transversal T2W, **b** early-phase dynamic contrast-enhanced T1W DIXON water only, **c** zoomed DWI with calculated b1400, and **d** ADC from b0 to b800

Figure 3 shows representative MR images of one of the patients. Of the 298 patients, 97 (33%) had PI-RADS 1–2, 44 (15%) had PI-RADS 3, and 157 (53%) had PI-RADS 4–5. 116 (39%) were not biopsied but referred to PSA monitoring or follow-up with MRI. Of these 116 non-biopsied patients, 85 (73%) had PI-RADS 1–2, 21 (19%) had PI-RADS 3, and 10 (9%) had PI-RADS 4–5. The reasons for not performing biopsy of the 10 patients with PI-RADS 4–5 were inflammation risk (*n* = 1), patient choice (*n* = 6), differential diagnosis of inflammation and rapid PSA decrease after MRI (*n* = 1), and MRI indicated a small, low-grade tumor combined with high age (*n* = 1) or comorbidity (*n* = 1).

Of the 298 patients, 108 had TRUS-guided biopsy and 74 had in-bore MRI biopsy, 15 of these after TRUS-guided biopsy (Fig. 1). In these 15 patients, 8 tumors were upgraded from ISUP 1 to 2, two from benign to ISUP 2, and five were unchanged.

**Fig. 4:**
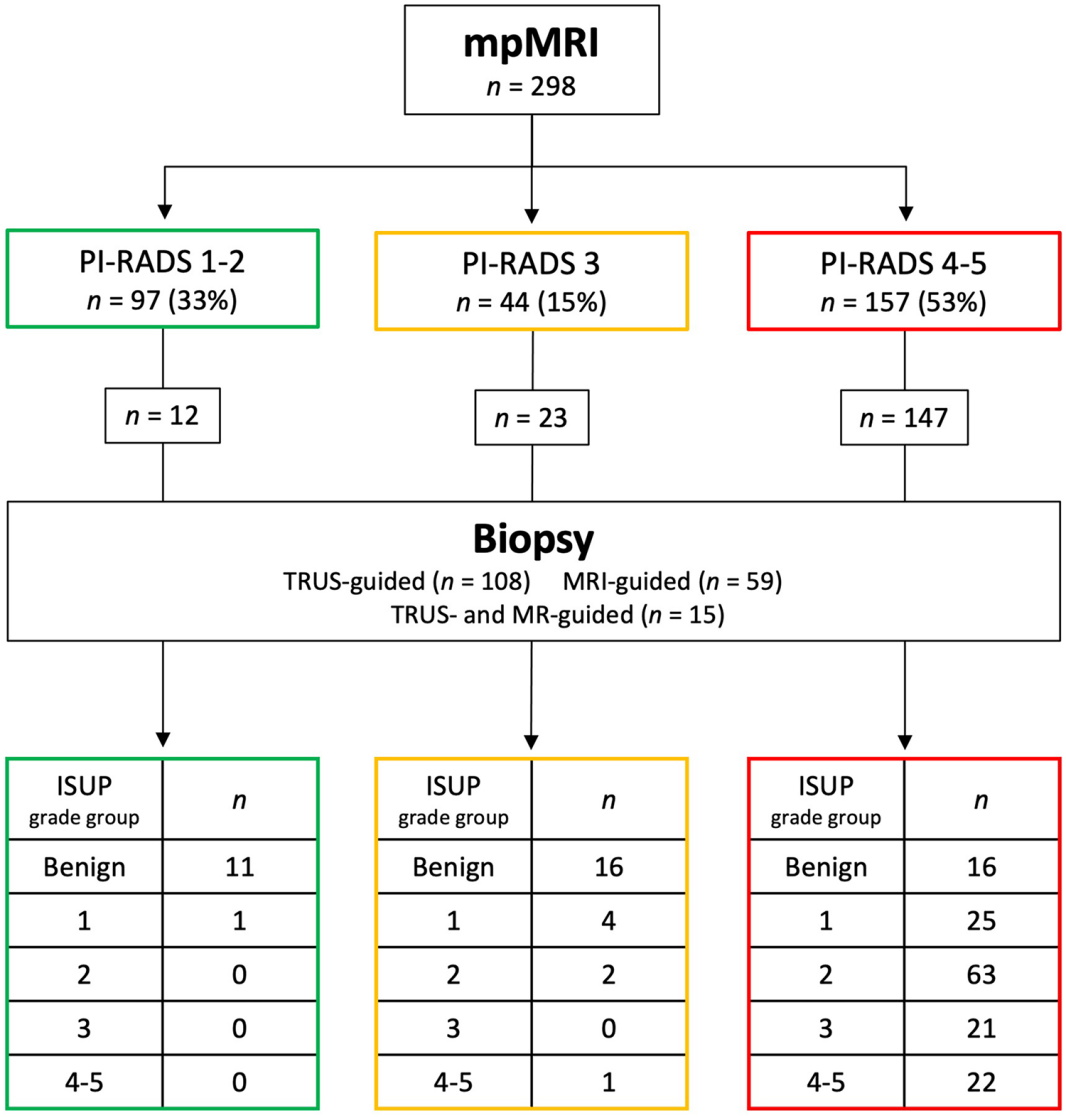
PI-RADS and biopsy findings for the 298 patients who were referred to our MRI-directed diagnostic pathway. *TRUS* transrectal ultrasound, *ISUP* International Society of Urological Pathology

The histopathological findings per patient as function of PI-RADS are summarized in Fig. 4. Prostate cancer was detected in 139 (46%) patients. Of these, 30 (22%) had ISUP 1, 65 (46%) had ISUP 2, 21 (15%) had ISUP 3, and 23 (16%) had ISUP 4–5. No cancer was found in 43 (24%) of the 182 biopsied patients. No clinically significant cancer was found in the PI-RADS 1–2 category and in only three of the 44 patients (7%) with PI-RADS 3. 42 of 43 high ISUP grade groups (≥ 3) were in the PI-RADS 4–5 category. 88 of 95 lower ISUP grade groups (≤ 2) were also in PI-RADS 4–5. No non-biopsied patients or PI-RADS 4–5 patients with benign biopsies were referred back to our hospital and diagnosed with clinically significant cancer during the 2.0–3.5-year-follow-up time.

Some patients had multiple lesions, in total 445 lesions were PI-RADS classified. The results *per lesion* are summarized in Online Resource 2.

All except three of the 111 PI-RADS 3–5 patients with TRUS-guided biopsies also had systematic biopsies covering MRI-negative regions. Eleven of these patients had positive biopsies from MRI-negative regions. All except one were lower than or equal in ISUP grade group than from the MRI-positive areas. One patient had a large PI-RADS 5 tumor harboring ISUP 4 on one side, with ISUP 4 and 5 in systematic biopsies on the other side.

Of the 298 patients, 38 had prostatectomy. Five were upgraded from the biopsies to the resected specimen: four from ISUP 2 to ISUP 3 and one from ISUP 1 to ISUP 3 (Online Resource 3).

## Discussion

In June 2019 the PI-RADS-steering committee advocated to implement a MRI-directed biopsy pathway enabling key diagnostic benefits to men with suspected prostate cancer [12]: that is to (a) reduce the number of patients who need prostate biopsy, (b) reduce the detection of clinically insignificant cancer, (c) increase detection of clinically significant cancers, and (d) improve risk stratification. Our two years of experience with a MRI-directed diagnostic pathway show that implementation in clinical routine in a non-university hospital is feasible and that the anticipated benefits can be obtained.

About 40% of our patients did not undergo biopsy and the majority of them had PI-RADS 1–2. This is in line with other ‘real-world’ results reported from the IMRIE study and by Sokhi et al. [23, 24]. Without template biopsies it is not possible to rule out that significant cancers have been overlooked. However, we had findings indicating that this is unlikely: of the 12 patients with PI-RADS 1–2 who underwent biopsy, none was diagnosed with clinically significant cancer (one had ISUP 1). Although limited follow-up time (2–3.5 years), none of the patients have been referred back to our hospital and diagnosed with clinically significant cancer.

Almost half of the PI-RADS 3 patients did not undergo immediate biopsy, an optional strategy in the algorithm proposed by the PI-RADS-steering committee. Of the biopsied PI-RADS 3 patients, two had ISUP 2 and one had ISUP 3. The number of PI-RADS 3 patients diagnosed with clinically significant cancer (7%) was lower than in other studies (12–31%) [5, 7, 10]. A possible explanation is that we used high-resolution DWI and DCE images making small lesions appear more discrete and with higher contrast between the lesion and the background. This could migrate some PI-RADS 3 lesions to PI-RADS 4.

The detection of ISUP 1 was 10% in our entire cohort. This is comparable to the results from MRI-directed strategies in multicenter trials and significantly lower than for the strategy using systematic biopsies in these trails [7, 10, 18]. In the MRI-targeted biopsy group we found 17% ISUP 1, also comparable to the results from these trials. Apparently, high-quality PI-RADS multiparametric MRI detects insignificant disease despite its aim not to do so [8]. One strategy to reduce this could be to lower the sensitivity of PI-RADS MRI, either by altering the interpretation criteria (PI-RADS) or reduce the sensitivity of the imaging. However, such adjustments can be expected to negatively impact detection of clinically significant cancer. An alternative strategy could be to incorporate parameters into PI-RADS and MRI that reflect tumor aggressiveness [25].

Our findings are similar to prospective multicenter trials documenting that a MRI-directed pathway in biopsy-naive patients can detect clinically significant cancer with high sensitivity [2, 7, 10, 18]. But is it safe to omit systematic biopsies? It has been shown that MRI misses some ISUP 2 [2, 26] and that systematic biopsies detect some of those [5, 17, 18]. The ultimate reference standard to detect upgrading is prostatectomy. In our cohort 38 patients underwent surgery and in five patients the histopathology was upgraded, four from ISUP 2 to 3 and one from ISUP 1 to 3. We also tried to detect missed cancers by looking for positive systematic biopsies from MRI-negative regions. We found 11 patients, and all except one were lower or equal in ISUP than from their MRI-positive regions.

The main limitation of our study was the lack of systematic template-based biopsies in all patients. The study was not designed to measure the accuracy of MRI, but to gain evidence of the safety of a MRI-directed diagnostic pathway implemented in ‘real-world’ clinical care. Second, the follow-up time of non-biopsied patients is limited. However, a longer follow-up time has a trade-off because it would be difficult to distinguish overlooked cancers from de novo transformed cancers. Third, the generalizability to other hospitals depends not only of the algorithm itself but also on the quality of all its steps. Especially the imaging protocol and patient preparations for the MRI may influence the generalizability as it varies widely in the literature and between institutions [16, 21, 27]. The strength of our study is that we attempted to optimize all steps through high image quality, experienced radiologists, high-precision biopsy targeting when required, and MDT composed of all involved disciplines: radiologist, urologist, oncologist, and pathologist.

In conclusion, our findings add to evidence that a MRI-directed diagnostic pathway can be safely established in a non-university hospital and delivers improved risk stratification, as advocated by the PI-RADS-steering committee [12]. Prostate cancer of uncertain clinical significance is still diagnosed.

## Supplementary Information

Below is the link to the electronic supplementary material.

Supplementary file1 (DOCX 20 KB)
Online Resource 1: The MRI scan protocol parameters

Supplementary file2 (DOCX 75 KB)
Online Resource 2: PI-RADS and biopsy findings per lesion. In one patient MRI detected two lesions, one assigned to PI-RADS 1-2 and one to PI-RADS 4-5. The PI-RADS 1-2 lesion was ISUP grade group 4 whereas the PI-RADS 4-5 was ISUP grade group 3. The lesion harboring ISUP grade group 4 was interpreted as PI-RADS 1-2, but the radiologist strongly recommended in-bore MR biopsy because of pronounced DCE-findings, thus both MRI lesions were biopsied. Because the per patient analysis showed only the lesion with highest PI-RADS score, the lesion with PI-RADS 1-2 and ISUP grade group 4 does not appear in Figure 3.

Supplementary file3 (JPG 1028 KB)
Online Resource 3: Ductal carcinoma (dotted ellipsoid) misinterpreted at T2W as a hyperplastic nodule bulging into the peripheral zone. Note the resemblance with the hyperplastic nodule (arrow) within the transitional zone.
