# Supplementary material for: Results from a PI-RADS-based MRI-directed diagnostic pathway for biopsy-naive patients in a non-university hospital
Source: Abdom Radiol (NY). 2021 Aug 20;46(12):5639–46. doi: 10.1007/s00261-021-03249-8 (PMC8590681; doi:10.1007/s00261-021-03249-8)
Supplement: Supplementary file 1 — Supplementary file1 (DOCX 20 KB) Online Resource 1: The MRI scan protocol parameters [file 261_2021_3249_MOESM1_ESM.docx]

**Online Resource 1: The MRI scan protocol parameters**

| Acquisition parameters | T2W TSE | DWI ZOOMit  (Oct 17-Dec 18) | DWI RESOLVE  (May 17- Sept 17) | T2W SPACE | DWI | DCE | T1W post Gd |
| --- | --- | --- | --- | --- | --- | --- | --- |
| ­­­­­­­­­MRI sequence | 2D SE | SE-EPI zoomed | SE-EPI | 3D SE | SE-EPI | 3D Spoiled GE-Dixon | 3D Spoiled GE-Dixon |
| Acquisition plane | sag/tra/cor | tra | tra | tra | tra | tra | cor |
| Echo time (ms) | 99 | 69 | 58 | 103 | 74 | 1.28 and 2.51 | 2.46 and 3.69 |
| Repetition time (ms) | 2000 (DE) | 3800 | 3640 | 1240 | 6800 | 4.27 | 5.45 |
| Flip angle | 150 |  |  | 125 |  | 10 | 10 |
| Slice thickness (mm)  Acquired  Interpolated | 3 | 3 | 3.3 | 1.42  1.0 | 5 | 3.30  3.00 | 2.36  1.50 |
| Slice gap (mm) | 0 | 0.8 | 1 | 0 | 1 | 0 | 0 |
| Number of excitations | 1/3/1 | 16 | 3 | 1.4 | 2 | 1 | 1 |
| In-plane resolution (mm x mm)  Acquired  Interpolated | 0.69x0.69/0.63x0.63/ 0.69x0.69  0.34x0.34/0.31x0.31 /0.34x0.34 | 1.69x1.69  0.85x0.85 | 1.70x1.70  1.70x1.70 | 0.93x0.88  0.44x0.44 | 2.6x2.6  1.3x1.3 | 1.38x1.31  0.66x0.66 | 0.90x0.90  0.45x0.45 |
| Echo train | 16/14/16 | 44 | 76 | 100 | 80 |  |  |
| Bandwidth (Hz/pixel) | 295 | 1500 | 806 | 651 | 2000 | 1080-890 | 870-810 |
| FOV (mm x mm) | 220x220/ 200x200/ 220x220 | 115 x 74 | 160x160 | 225x225 | 260x208 | 210x210 | 345x345 |
| Matrix size (pixels x pixels)  Acquired  Interpolated | 320 x 320  640 x 640 | 68x68  136x136 | 94x94  NA | 256x243  512x512 | 100x100  200x200 | 160x152  320x320 | 384x384  768x768 |
| Parallell imaging  acceleration factor^a^ | 1/2/1 | 1 | 2 | 3 | 1 | 2x2 | 2x2 |
| Motion correction | no | no | no | no | no | yes^b^ | no |
| b-values (s/mm^2^) | NA | 0-800;  calculated b1400 | 0-800;  calculated b1500 | NA | 0-800 | NA | NA |
| Time resolution (sec) | NA | NA | NA | NA | NA | 10 | NA |
| Acquisition time | 2:46 / 4:42 / 2:46 | 5:17 | 6:35 | 5:19 | 1:30 | 4:34 | 1:28 |

T2W = T2 Weighted MRI, TSE = Turbo Spin Echo, DWI = Diffusion Weighted Imaging, RESOLVE = REadout Segmentation Of Long Variable Echo train, DCE = Dynamic Contrast-Enhanced MRI, Gd = Gadolinium, SE = Spin Echo, EPI = Echo Planar Imaging, GE = Gradient Echo, DE = Driven Equilibrium, FOV = Field Of View,

^a^Parallel imaging acceleration factor in more than one direction are given as (NxN), ^b^Performed using SyngoVia (Siemens).
