# Supplementary material for: Results from a PI-RADS-based MRI-directed diagnostic pathway for biopsy-naive patients in a non-university hospital
Source: Abdom Radiol (NY). 2021 Aug 20;46(12):5639–46. doi: 10.1007/s00261-021-03249-8 (PMC8590681; doi:10.1007/s00261-021-03249-8)
Supplement: Supplementary file 2 — Supplementary file2 (DOCX 75 KB) Online Resource 2: PI-RADS and biopsy findings per lesion. In one patient MRI detected two lesions, one assigned to PI-RADS 1-2 and one to PI-RADS 4-5. The PI-RADS 1-2 lesion was ISUP grade group 4 whereas the PI-RADS 4-5 was ISUP grade group 3. The lesion harboring ISUP grade group 4 was interpreted as PI-RADS 1-2, but the radiologist strongly recommended in-bore MR biopsy because of pronounced DCE-findings, thus both MRI lesions were biopsied. Because the per patient analysis showed only the lesion with highest PI-RADS score, the lesion with PI-RADS 1-2 and ISUP grade group 4 does not appear in Figure 3. [file 261_2021_3249_MOESM2_ESM.docx]

**Supplementary Figure 1:** PI-RADS and biopsy findings per lesion. In one patient MRI detected two lesions, one assigned to PI-RADS 1-2 and one to PI-RADS 4-5. The PI-RADS 1-2 lesion was ISUP grade group 4 whereas the PI-RADS 4-5 was ISUP grade group 3. The lesion harboring ISUP grade group 4 was interpreted as PI-RADS 1-2, but the radiologist strongly recommended in-bore MR biopsy because of pronounced DCE-findings, thus both MRI lesions were biopsied. Because the per patient analysis showed only the lesion with highest PI-RADS score, the lesion with PI-RADS 1-2 and ISUP grade group 4 did not appear in Figure 3.
