# Supplementary figures and images for: Results from a PI-RADS-based MRI-directed diagnostic pathway for biopsy-naive patients in a non-university hospital
Source: Abdom Radiol (NY). 2021 Aug 20;46(12):5639–46. doi: 10.1007/s00261-021-03249-8 (PMC8590681; doi:10.1007/s00261-021-03249-8)

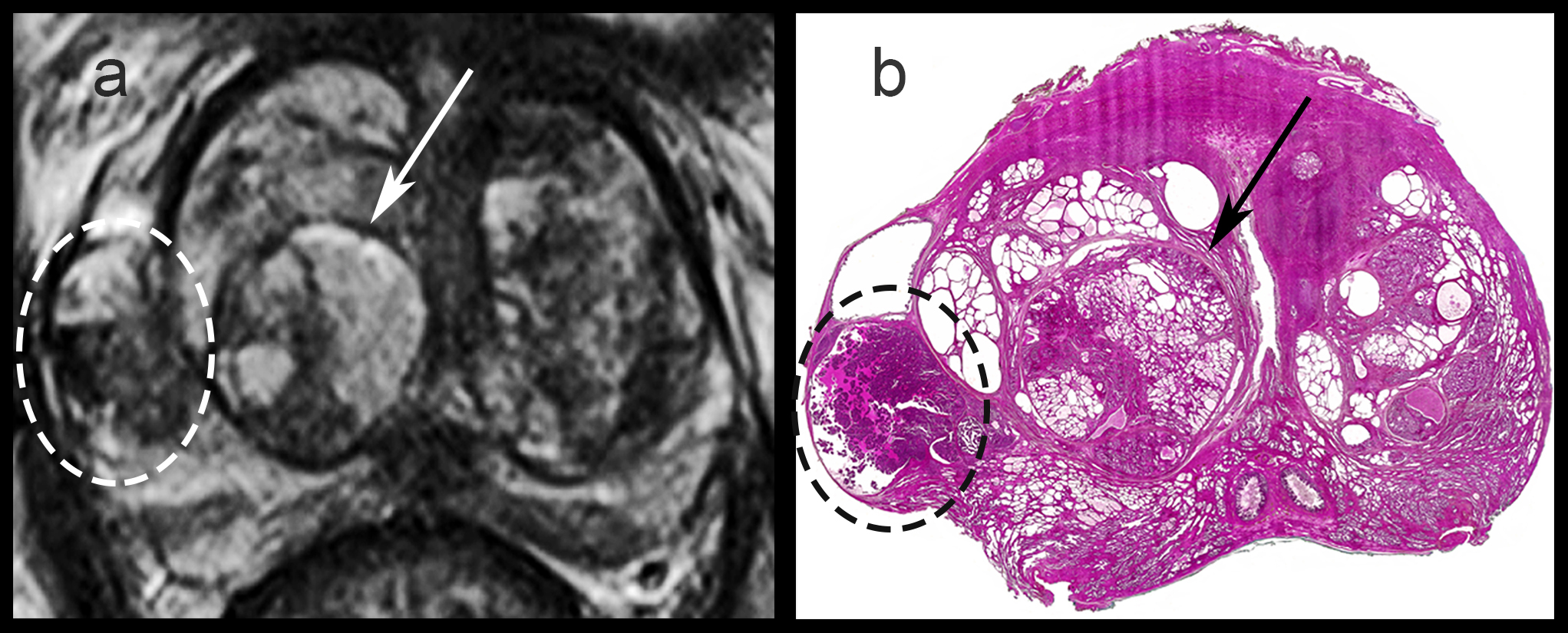

Supplement: Supplementary file 3 — Supplementary file3 (JPG 1028 KB) Online Resource 3: Ductal carcinoma (dotted ellipsoid) misinterpreted at T2W as a hyperplastic nodule bulging into the peripheral zone. Note the resemblance with the hyperplastic nodule (arrow) within the transitional zone. [file 261_2021_3249_MOESM3_ESM.jpg]
